# A comparison of distributed machine learning methods for the support of “many labs” collaborations in computational modeling of decision making

**DOI:** 10.3389/fpsyg.2022.943198

**Published:** 2022-08-25

**Authors:** Lili Zhang, Himanshu Vashisht, Andrey Totev, Nam Trinh, Tomas Ward

**Affiliations:** ^1^School of Computing, Dublin City University, Dublin, Ireland; ^2^Insight Science Foundation Ireland Research Centre for Data Analytics, Dublin, Ireland; ^3^In the Wild Research Limited, Dublin, Ireland

**Keywords:** deep learning, decision-making, distributed learning, federated learning, data privacy

## Abstract

Deep learning models are powerful tools for representing the complex learning processes and decision-making strategies used by humans. Such neural network models make fewer assumptions about the underlying mechanisms thus providing experimental flexibility in terms of applicability. However, this comes at the cost of involving a larger number of parameters requiring significantly more data for effective learning. This presents practical challenges given that most cognitive experiments involve relatively small numbers of subjects. Laboratory collaborations are a natural way to increase overall dataset size. However, data sharing barriers between laboratories as necessitated by data protection regulations encourage the search for alternative methods to enable collaborative data science. Distributed learning, especially federated learning (FL), which supports the preservation of data privacy, is a promising method for addressing this issue. To verify the reliability and feasibility of applying FL to train neural networks models used in the characterization of decision making, we conducted experiments on a real-world, many-labs data pool including experiment data-sets from ten independent studies. The performance of single models trained on single laboratory data-sets was poor. This unsurprising finding supports the need for laboratory collaboration to train more reliable models. To that end we evaluated four collaborative approaches. The first approach represents conventional centralized learning (CL-based) and is the optimal approach but requires complete sharing of data which we wish to avoid. The results however establish a benchmark for the other three approaches, federated learning (FL-based), incremental learning (IL-based), and cyclic incremental learning (CIL-based). We evaluate these approaches in terms of prediction accuracy and capacity to characterize human decision-making strategies. The FL-based model achieves performance most comparable to that of the CL-based model. This indicates that FL has value in scaling data science methods to data collected in computational modeling contexts when data sharing is not convenient, practical or permissible.

## 1. Introduction

One of the most important tasks in cognitive psychology and behavioral science is to understand and explain how people think, infer and process information. This is generally achieved through observing their behavior under experimental settings. In the field of human decision making, where we evaluate how people make decisions based on learning from experience, we can either analyze the process by which information is itself processed (Ratcliff and McKoon, 2008; Summerfield and Tsetsos, 2012) or investigate how the expectation values (the decision's outcome multiplied by the probability of that decision) of options are learned and updated through experience (Rescorla, 1972; Dayan and Niv, 2008). In such approaches, we use cognitive models that specify a set of assumptions about the underlying learning processes used by the subjects, thus generally requiring manual engineering with an iterative process to examine the consistency between the hypothesis and the empirical data (Dezfouli et al., 2019b). The approach may fail if subjects adopt a completely different strategy during decision making. Deep Learning (DL) models, especially Recurrent Neural Network (RNN) models, present an alternative approach to the characterization of human decision-making behavior. They can automatically capture behavioral trends exhibited by subjects without strong assumptions about the mathematical structure of the underlying process through taking advantage of their data-driven design and higher capacity for representing complex computational processes (Dezfouli et al., 2019a,b; Fintz et al., 2021).

Unfortunately, this does not come for free since modern DL models involve the learning of a large number of parameters which in turn requires large amounts of diverse data for effective performance. For example, in a recent study (Zech et al., 2018), it was found that DL models overfit when trained on small and biased institutional data and generalized poorly on data from institutions whose data were not seen during training. Although the data used in that study comprised of medical images rather than behavioral data, it is a good example and a warning that DL models need to be carefully trained. Efforts must be made to minimize the introduction of confounding factors associated with experimental biases which can dominate the training process and obscure the relationship of predictions to the underlying targeted pathology. Such models may perform with good accuracy when testing against held-out data from the same experiment, but may not generalize well to the same experiments conducted in other studies. A natural way to increase both data size and diversity is through collaborative learning, in which many laboratories cooperate and contribute data to train a global model together. It is an effective approach that has the potential to solve the limitation in most cognitive studies where the number of subjects is adequate for the modeling approach originally intended but then becomes inadequate when the same data is used to train new models involving a larger number of parameters. Furthermore, one of the significant challenges in computational psychiatry (Huys et al., 2016), i.e., translating advances in understanding the cognitive biases of people with mental illness into improvements in clinical practice, will also benefit from the pattern of multi-experiment collaboration, given that the scale of current cognitive studies is not sufficient for developing robust models that satisfy clinical needs.

One of the most conventional options for conducting “many-labs” analysis requires different laboratories to share subjects' data to a centralized location for model training (i.e., centralized training). However, in practice, data sharing is not easy, especially when involving larger number of laboratories from different legal jurisdictions, due to privacy, ethical, and data regulation barriers (Voigt and Von dem Bussche, 2017). Consequently, information coming from various populations worldwide remains distributed in isolation, across multiple laboratories, and we lose the benefits that could accrue from combining data. A solution for addressing this to some degree is distributed model training, in which model parameters instead of individual identifiable information are shared among laboratories. One approach for distributed training is unparallel, or as we will refer to it here—incremental distributed learning (IL) (Sheller et al., 2018). With this approach each laboratory trains the model and passes the learned model parameters to the next laboratory for training on its data, until all have trained the model once. Another approach is an extension of incremental distributed learning, called cyclic incremental distributed learning (CIL). This involves the repetition of the incremental learning process multiple times, i.e., fixing the number of training iterations at each laboratory and cycling repeatedly through the laboratories. Chang et al. (2018) explored these two distributed training approaches using medical imaging data and found the cyclic approach performed comparably to centralized training, suggesting that sharing data may not always be necessary to build deep learning models for patient imaging data. The incremental distributed learning approaches, however, have been criticized for introducing the problem of “catastrophic forgetting” (French, 1999), where the trained model strongly favors the data it has most recently seen. Nevertheless, the repeated cycles and limited iterations per institution performed in cyclic incremental learning enable it to make gradual progress, despite the forgetting issue, resulting in better models than the first distributed training approach.

Federated learning (FL) can be considered another distributed machine learning paradigm seeking to directly address the problem of data governance and privacy. It was first introduced by Google AI (Konečnỳ et al., 2016) to allow mobile devices collaboratively learn machine learning models without sharing data from the devices. It was applied very successfully in training Google's autocomplete keyboard application (Hard et al., 2018). Institutions or laboratories can also be viewed as “devices” in the context of FL and therefore FL has emerged as a promising strategy in scenarios where, for example, hospitals operate under strict privacy practices and may face legal, administrative, or ethical constraints that require data to remain local. Different from the unparallel approaches, FL is a data parallel training process, in which multiple collaborators train a DL model simultaneously (each on their own data in parallel) and then send their model parameters to a central sever where these are aggregated into a global model. The central sever then sends the global model to all collaborators for further training. Each iteration of this process, i.e., parallel training, parameter update, and distribution of global parameters, is referred to as a federated round. Sheller et al. (Sheller et al., 2018, 2020) compared FL and IL approaches on imaging datasets and unsurprisingly, the basic IL performed the poorest compared to FL and CIL. While cyclic incremental learning may seem a simpler alternative, given that implementation of FL depends on a set of key challenges (Li et al., 2020a), e.g., communication efficiency, which is outside the scope of this article, it required additional validation steps at the end of each cycle, which are basically as complex as the synchronization logic of FL, to achieve comparable results to FL paradigm. Critically, CIL was less stable than FL, resulting in an inferior alternative. The degree to which the laboratory datasets used for distributed learning are independent and identically distributed (IID) can have a significant influence on the learning performance compared to centralized learning (Sheller et al., 2020) as deep learning models rely on Stochastic Gradient Decent (SGD) algorithm and the IID samples can ensure that the stochastic gradient is an unbiased estimate of the full gradient (Rakhlin et al., 2011). However, in practice, it is unrealistic to assume that the local data of each laboratory is always IID, which is also a statistical challenge for applying federated learning. It was found in Zhao et al. (2018) that the accuracy of a federated learning model trained for image classification was reduced by up to 55% depending on how much institutional bias or degree of non-IID they introduced while dividing a single dataset into hypothetical institutions. The institutions and institutional biases in their paper were created artificially by defining hypothetical clients and assigning various number of samples to the clients. However, the results of applying the distributed approach on artificially partitioned hypothetical institutional datasets may fail to account for how real-world clients biases impact distributed learning paradigm, a point that has been argued in Sheller et al. (2020). Consequently, this also motivated this paper in which we experiment with real-world “many-labs” datasets.

In this study, we examined and compared the reliability and feasibility of applying distributed learning strategies to neural network models of human decision-making processes. This is demonstrated using a real-world “many-labs” study comprising 10 different laboratories (Steingroever et al., 2015). Subjects in all studies were healthy participants and completed a computerized version of the Iowa Gambling Task (IGT), which is one of the most widely used tasks measuring human decision making under uncertainty in an experimental context. Distributed learning paradigms, especially FL, have been examined in various application scenarios including electrocardiogram datasets (Lee and Shin, 2020), FMRI datasets (Li et al., 2020b; Sheller et al., 2020), mobile health datasets (Liu et al., 2021), and biomedical datasets (Dankar et al., 2019). Nevertheless, among this literature none of them has reported on the use of distributed learning paradigms on trial-by-trial decision making data originating from different laboratories, even though such experiments have become important methods for investigating human behavior. We first trained single laboratory models for each laboratory in the data pool and then evaluated each of these models against held-out testing datasets from each laboratory defined prior to model training. This experiment is used to demonstrate the need for numerous and diverse data for training a robust deep learning model. Secondly, we illustrated the benefits of “many-labs” collaboration and the superiority of federated learning compared to incremental learning and cyclic incremental learning. Thus, one centralized model and several distributed models were trained for this purpose. On-policy and off-policy simulations were then conducted to compare the performance of the three distributed learning strategies. Together these simulation approaches allow us to evaluate comprehensively how well the models learn the characteristics of human decision-making. Apart from assessing the relative performance of distributed learning compared to the centralized learning paradigm, these simulation analyses also allow us to reveal the underlying learning mechanisms and decision-making strategies used by the healthy subjects.

## 2. Materials and methods

### 2.1. The IGT

The IGT was originally developed to study the decision making deficits of ventromedial prefrontal cortex patients (Bechara et al., 1994). Over two decades, it has been one of the most widely used neuropsychological paradigms for simulating complex and experience-based decision-making (Steingroever et al., 2018). Participants in the IGT are initially given *e*2,000 virtual money and presented with four decks of cards labeled A, B, C, and D. Each card in these decks can generate rewards, and sometimes cause losses. Participants have to choose one card from these four decks consecutively, until the task terminates automatically after a fixed number of trials have been reached. In each trial, feedback on rewards and losses for their choice and the running tally over all trials so far are provided to the participants, but no information is given regarding how many trials they will play and how many trials they have completed during the task. Participants are instructed that they can choose cards from any deck and they can switch decks at any time. They are also told to make as much money as possible by minimizing losses.

### 2.2. The IGT dataset

The data pool (*N* = 617) we used in this study derives from 10 studies assessing performance of healthy participants (i.e., without any known neurological impairments) on the IGT (Steingroever et al., 2015). It involves a broad range of healthy populations aging from 10 to 88 with various education backgrounds and social status. Participants completed a computerized version of the IGT consisting of 95, 100 or 150 trials. All included studies used (a variant of) the traditional IGT payoff scheme or the payoff scheme introduced by Bechara and Damasio (2002). The variations are described in the original paper and primarily concern the time-varying nature of rewards and losses across the trials. For example, the loss associated with deck C in the traditional payoff scheme varies across trials, whereas the deck C loss in the variant scheme here is held constant. More details about the payoff schemes of each variant can be found in Steingroever et al. (2015).

Since the RNN model we used for predicting subjects' actions required us to have inputs with the same size, the trial-by-trial decision sequences of all subjects were truncated according to the length of the smallest sequence, i.e., 95 trials. In other words, for those subjects who completed 100 or 150 trials of the IGT, only the first 95 trials were used for training or testing the model. Each subject is an abstract data point (Jung, 2022) and each data point is a sequence of decision-making choices that contains 95 trials. The features of the data points are the observed choices so far and the labels, which are also what we want to predict, are the choices in the next trials. The sample size of each laboratory, which we will refer to lab 1-10, are 15, 162, 19, 40, 70, 25, 153, 35, 57, and 41, respectively.

This dataset is reused here because, (1) first of all, participants in all studies were healthy subjects and they all completed the same decision making task—IGT, which makes it feasible to train a collaborative model for all laboratories; (2) the subjects from 10 studies were coming from different backgrounds with various ages and female proportions, thus, conducting multi-laboratory analysis is a natural operation to increase data size and diversity and improve the performance of the model; (3) it created a perfect real-world scenario where the local data of each lab is potentially non-IID and biased, thus it is suitable for testing out the reliability and effectiveness of distributed learning paradigms, especially federated learning, that are sensitive to the data distribution. Each laboratory can be seen as a “device” in the context of federated learning and the data assignments for the clients in FL can be matched with the real-world data distributions, such that all subjects from the same study are assigned to the same client; (4) last but not least, we can more thoroughly investigate how healthy subjects learn and behave on the IGT using deep learning models, which as stated previously, are promising in the area of decision making modeling because of their higher capability of learning from data in a less model-constrained manner.

### 2.3. Applying RNN to the IGT

**Figure 1:**
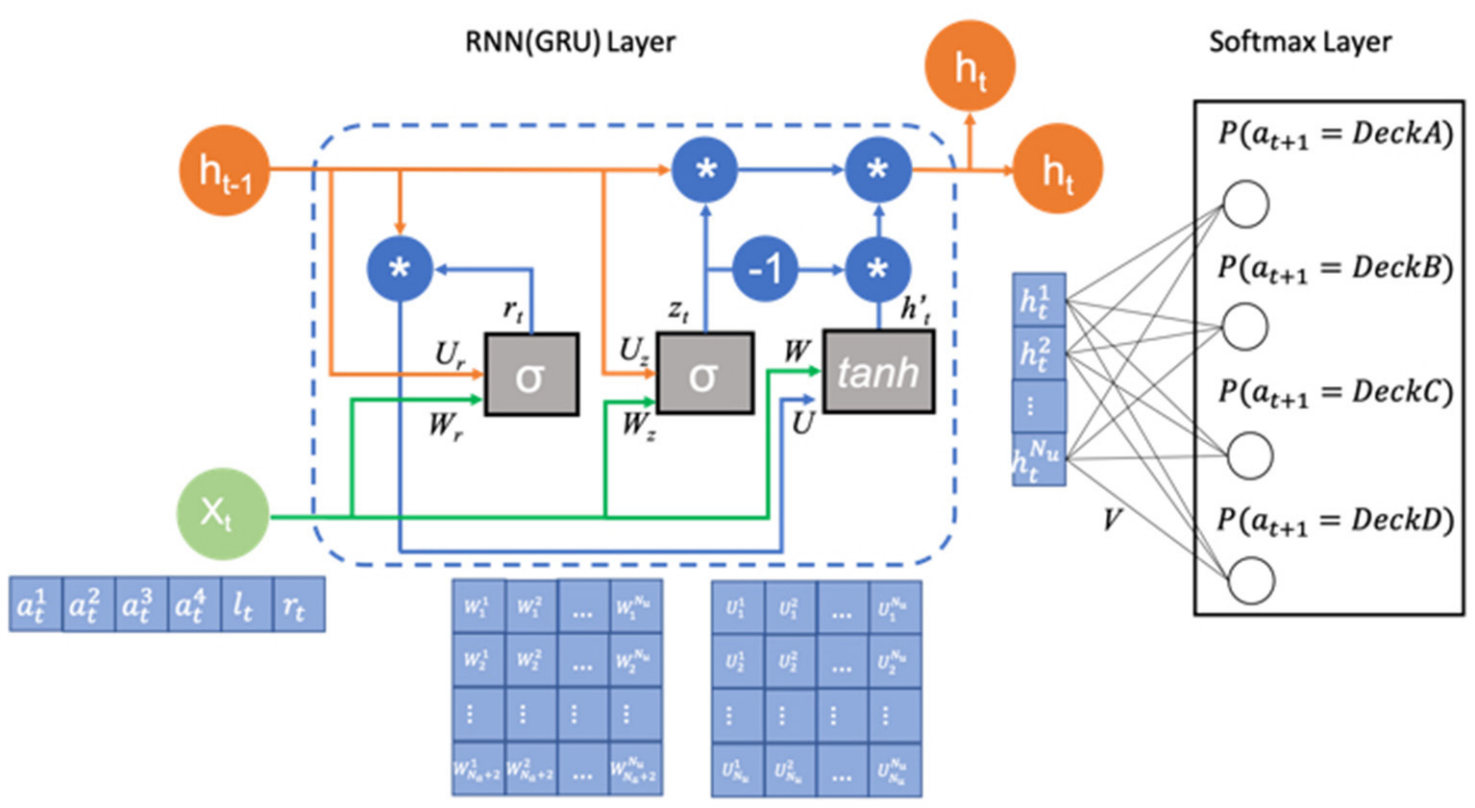
Architecture of the RNN model. The model has a GRU layer **(the left part)**, which receives the previous action (the action is one-hot coded), loss and reward *x*_*t*_ and previous hidden states *h*_*t*−1_ and a softmax layer **(the right part)**, which outputs the probability of selecting each deck on the next trial. The GRU layer is composed of two gates, i.e., an update gate and a reset gate, containing a set of weight parameters {*W*_*r*_, *U*_*r*_, *b*_*r*_, *W*_*z*_, *U*_*z*_, *b*_*z*_*W, U, b*_*hw*_, *b*_*hU*_}(*b*_*r*_, *b*_*z*_, *b*_*hW*_, and *b*_*hU*_ are not shown for simplicity). The output of the cells of the GRU layer *h*_*t*_ are connected to the softmax layer using black lines. The weight parameters in the softmax layer are represented as *V*.

In order to model the data produced during the IGT, we make use of state-of-the-art neural network approaches (Dezfouli et al., 2019b), such as RNNs. The RNN model we used is composed of a GRU layer and an output softmax layer with four nodes corresponding to the four choices for the different decks in the IGT. The inputs to the GRU layer are the previous choices made by the subject along with the rewards and losses they received after making that choice. The softmax output are the probabilities of choosing each deck option and sum to 1. The architecture of the model is shown in Figure 1.

It is known that the basic RNNs are inefficient in solving problems that require the learning of long-term temporal dependencies because of the gradient vanishing problem (Gers et al., 2000). If a sequence is long enough, which is the case of our choice sequences that include at least 95 trials, they'll fail to convey information (the deck preferences) from earlier trials to later ones. LSTM (Hochreiter and Schmidhuber, 1997) and GRU (Cho et al., 2014) networks were created as the solution to this short-term memory issue. In most cases, the two approaches yield comparable performance. It is often the case that the tuning of hyperparameters may be more important than choosing the networks. We chose the GRU here in this paper since it has fewer parameters and can be trained faster. We believe this to be a suitable choice given that we have a relatively small dataset with moderately long sequences.

The GRU layer is composed of a set of hidden units (*N*_*u*_). Each unit is associated with a unit output denoted by $h_{t}^{k}$ for cell *k* at time *t*. Let's define $h_{t}={\left[h_{t}^{1},h_{t}^{2},...h_{t}^{N_{u}}\right]}^{T}$ ($h_{t}\in R^{N_{u}}$) as a vector containing all the cell outputs at time *t*. They are initialized with the value of zero and are updated after receiving each time step input. $x_{t}\in R^{N_{c}+2}$ (*N*_*c*_ is the number of choices, which is 4 for the IGT) is a vector containing inputs to the network at time *t*, i.e., the action *c*_*t*_ coded using one-hot representation and the reward *r*_*t*_ and loss *l*_*t*_ received after taking action. There are two gates in GRU network, i.e., a reset gate and update gate. The update gate helps the model to determine how much of the past information needs to be passed along to the future. The output of this gate *z*_*t*_ is a linear combination of the input vector of the current time step *x*_*t*_ and the previous cell output going through the sigmoid function.


(1) $$\begin{array}{r} z_{t}=\sigma \left(W_{z}x_{t}+U_{z}h_{t-1}+b_{z}\right) \end{array}$$

The reset gate is used to decide how much past information is forgotten. The formula is the same as that used for the update gate. The difference arises from the weights and the gate's usage, which we will examine later in this section.


(2) $$\begin{array}{r} r_{t}=\sigma \left(W_{r}x_{t}+U_{r}h_{t-1}+b_{r}\right) \end{array}$$

The output of the reset gate is then passed through the current memory content, in which the reset gate is used to store the relevant information from the past.


(3) $$\begin{array}{r} h_{t}^{\prime }=tanh\left(Wx_{t}+b_{hW}+r_{t}⊙Uh_{t-1}+b_{hU}\right) \end{array}$$

In the last step, the network needs to calculate the *h*_*t*_ vector which holds information for the current unit and passes it down to the network. In order to do this, the update gate is needed. It determines what to collect from the current memory content $h_{t}^{\prime }$ and what to take from previous steps *h*_*t*−1_. The parameters of the GRU layer include $W_{z},W_{r},W\in R^{N_{u}\times \left(N_{a}+2\right)}$, $U_{z},U_{r},U\in R^{N_{u}\times N_{u}}$, and $b_{z},b_{r},b_{hW},b_{hU}\in R^{N_{u}}$.


(4) $$\begin{array}{c} h_{t}=z_{t}⊙h_{t-1}+(1-z_{t})⊙h_{t}^{\prime } \end{array}$$

The softmax layer takes outputs from the GRU layer (*h*_*t*_) as its inputs and outputs the probability of choosing each action. The parameter of the softmax layer is $V\in R^{N_{u}\times N_{a}}$. The parameters of the RNN model will be Θ = {*V, W*_*z*_, *W*_*r*_, *W, U*_*z*_, *U*_*r*_, *U, b*_*z*_, *b*_*r*_, *b*_*hW*_, *b*_*hU*_}.

The RNN model was trained using the maximum-likehood (ML) method and Categorical Cross-Entropy Loss summed over all subjects on all trials:


(5) $$\begin{array}{r} Loss=-\overset{S}{\underset{s=1}{\sum }}\overset{T}{\underset{t=1}{\sum }}y_{s_{t}}log\left({ŷ}_{s_{t}}\right) \end{array}$$

The model was implemented in TensorFlow (Abadi et al., 2016) and optimization was based on the use of the Adam optimizer (Kingma and Ba, 2014).

### 2.4. The structure of the four learning paradigms

**Figure 2:**
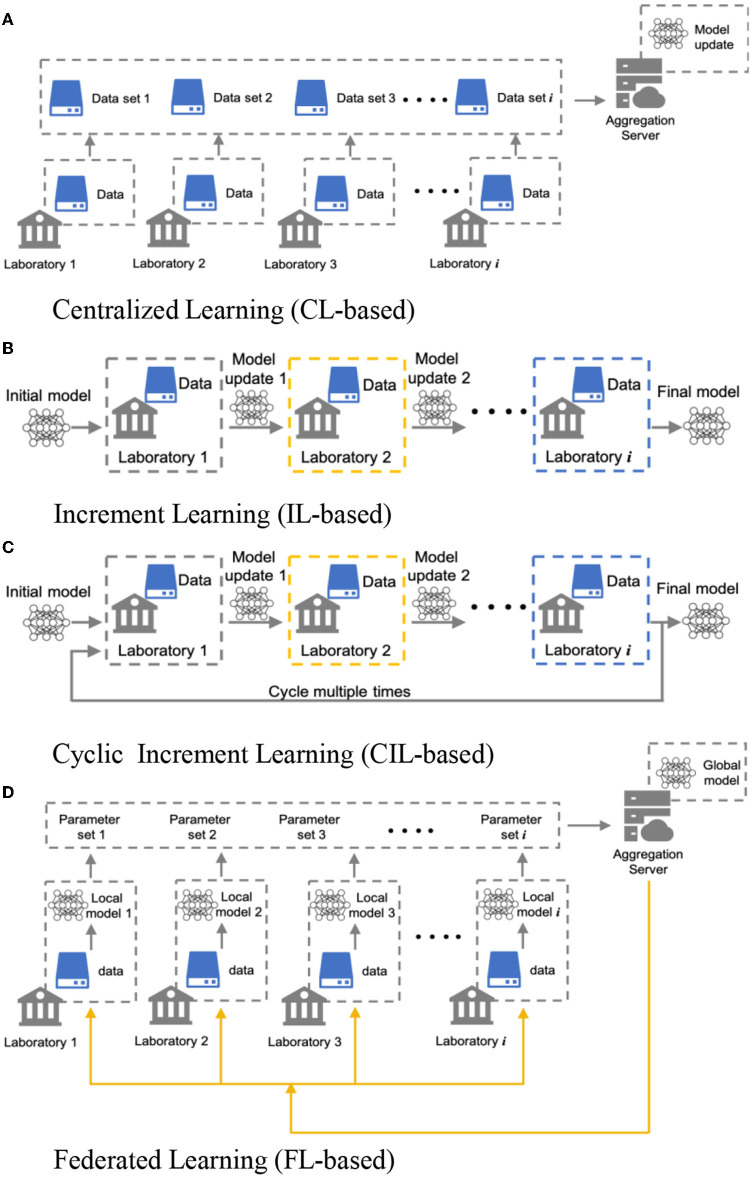
Architectures of four collaborative learning methods for multi-laboratory collaboration. The most straightforward method **(A)** is based on centralized data sharing, i.e., requiring that every laboratory share data to a central node and aggregating to train a model; **(B)** Incremental Learning, in which each laboratory trains the model and passes the learned model parameters to the next laboratory for training on its data, until all have trained the model once. **(C)** Cyclic incremental learning, which involves the repetition of the incremental learning process multiple times. **(D)** Federated learning, in which all laboratories train the same model at the same time and update their model parameters to a central sever where it is used to create a global model.

Figure 2 demonstrates the architectures of the four collaborative learning strategies. The first CL-based strategy is the most straightforward approach. Here, data is shared directly among laboratories. However, as stated above, data sharing is not always applicable because of data protection and regulation purposes, which makes this collaboration paradigm difficult to achieve in the real world. Distributed learning, i.e., the following three strategies in Figure 2, makes it possible to collaborate through sharing the model parameters rather than the more sensitive raw data. In the CL-based strategy, each laboratory trains the model once with their own data and passes the model parameters to the next laboratory to continue the training. The training process is finished when all laboratories train the model once. In the CIL-based strategy, this process can be repeated several times. In the FL-based strategy, all the laboratories train the model with their data simultaneously and share their model parameters to a central server to build a global model. All laboratories will receive the parameters of the global model and update their parameters to match the global model with certain probabilities. We expect CL-based model will behave the best in terms of prediction accuracy and capturing characteristics of human decision-making strategies among the four collaborative models, while the other three distributed learning paradigms will sacrifice competitiveness to different extents in exchange for preservation of data privacy. Thus, the CL-based model will serve as the baseline model against which comparisons are made with the distributed models. All of the learning paradigms are implemented in Python 3.7. The codes for replicating the results in this paper are available on OSF (osf.io/9ekr5/).

## 3. Results

### 3.1. Experimental settings

First of all, each laboratory dataset was divided into training and testing datasets with 80% of the data points for the former and the remaining 20% for the latter. Subjects were not mixed across training and testing sets. Two experiments are conducted with these datasets.

- The first experiment is designed to establish the need for bigger and more diverse data to train a reliable and robust RNN model that could predict human actions with reasonable accuracy. In this experiment, we train a single RNN model on the training dataset for each laboratory and evaluate each of these models against testing datasets from each laboratory. In total, we will train 10 independent RNN models, one for each separate laboratory, and evaluate their generalization performance both on the testing dataset of their own laboratory and testing dataset from the other laboratories.
- In the second experiment, we seek to demonstrate the improvements of collaborative models compared to single models and also to make a comparison between the centralized learning model and distributed learning models. Eight collaborative models will be trained. The collaborative models are trained directly or indirectly with bigger and more diverse datasets depending on the collaboration methods imposed between the laboratories, i.e., centralized learning (CL-based), incremental learning (IL-based), cyclic incremental learning (CIL-based), and federated learning (FL-based) paradigms. In incremental and cyclic incremental learning, three different orders of training the model are performed, i.e., smallest sample first, largest sample first and random order, yielding three models for these two learning paradigms.

The hyperparameters for training the centralized model are set as follows: batch size 32; maximum epochs 250; learning rate 0.02; number of hidden cells 10. To create an equal playing ground for the centralized learning and distributed learning paradigms, we need to guarantee that all models see exactly the same number of samples in the whole training process. Accordingly, the number of communication and the epochs per round for FL paradigm are set as 5 and 50, respectively. The maximum epochs for each laboratory is set as 250 for IL-based model. For the CL-based model, the frequency of weight transfer is every 50 epochs, thus the number of cycles is 5. The learning rate and the number of hidden cells are set as the same values for all models. The batch size for the distributed learning paradigms is the size of each laboratory sample. For the single models, two hyperparameters, the number of cells and epochs, are tuned *via* 10-fold cross validation. Please see Table 1, in which the parameter settings for each model are listed. More details about hyperparameter tuning are provided in the Supplementary material.

**Table 1:** Parameter setting for centralized and distributed learning models.

**Model**	**Batch size**	**Number of epochs**	**Learning rate**	**Number of hidden cells**	**Number of cycles**	**Communication rounds**
CL-based	50	250	0.02	10	N/A	N/A
IL-based	Size of each lab training sets	250	0.02	10	N/A	N/A
CIL-based	Size of each lab training sets	50	0.02	10	5	N/A
FL-based	Size of each lab training sets	50	0.02	10	N/A	5

### 3.2. The need for more numerous and diverse data

**Figure 3:**
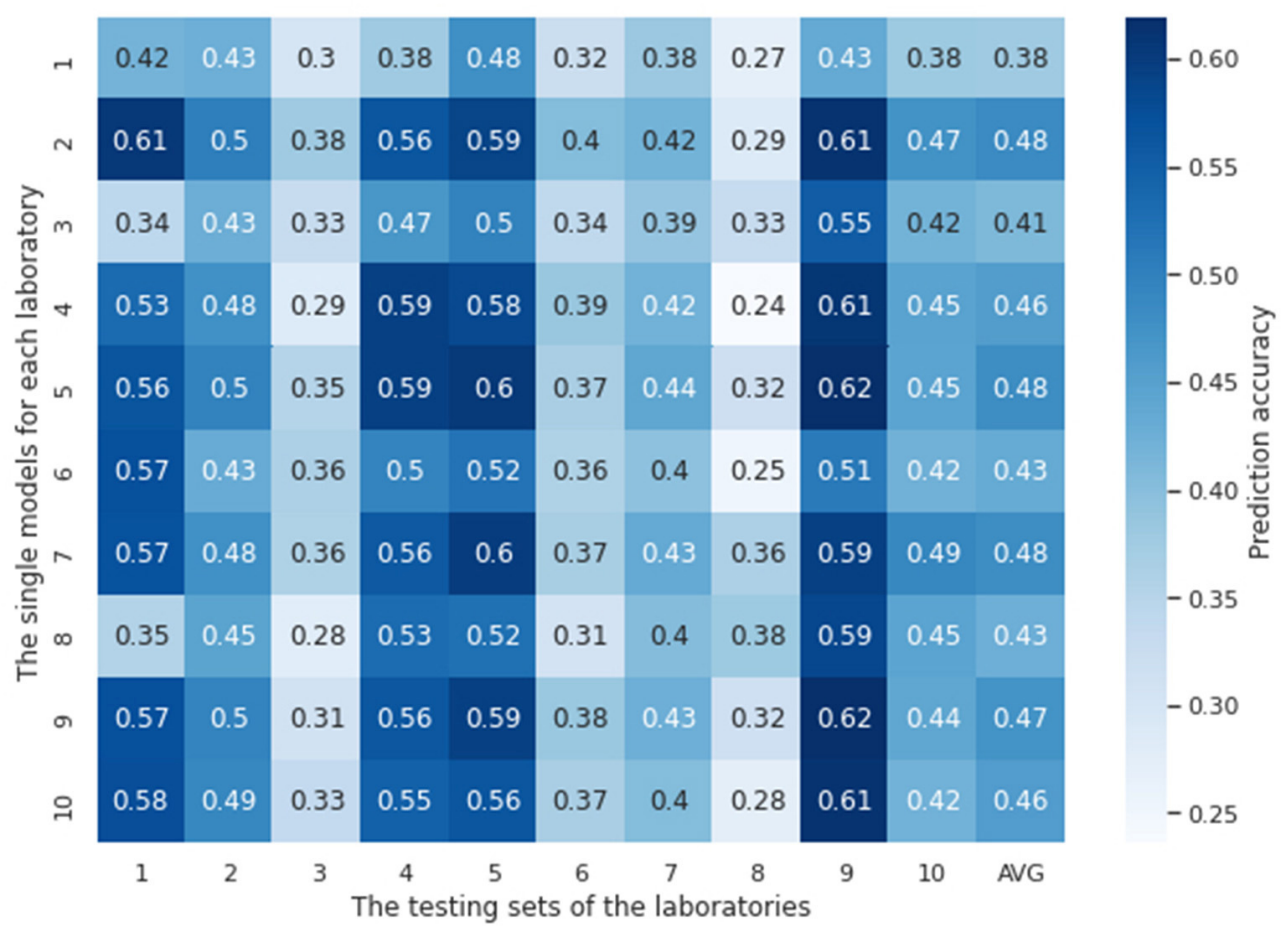
Accuracy of single lab models tested against testing datasets from each of the laboratories. The vertical axis represents models trained on a single laboratory dataset, and the horizontal axis represents the testing dataset of each independent laboratory. AVG is the average accuracy of each laboratory model performance over all laboratories. Overall, the prediction accuracy of each single model on the testing sets, no matter from their own laboratory or other laboratories, are not satisfying. Some of them are lower than the chance probability 25%.

**Figure 4:**
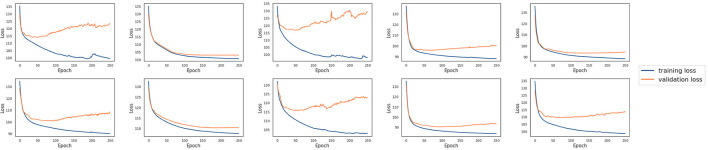
The training and validation loss of the 10 single models for each laboratory. The training loss decreases over time, whereas the validation loss starts increasing shortly after the first 10 or 20 epochs.

In this experiment, ten single models were trained with the tuned hyperparameters (see Supplementary material for details about how they are tuned) using the training datasets from each laboratory, respectively. These models were then evaluated using the testing datasets available from each laboratory. Figure 3 shows ten single models' testing results both against their own testing datasets and testing datasets of other laboratories. The chance probability of predicting the next action taken by the subjects correctly is 25% since there are four options in the IGT. However, the prediction accuracy of several single models on some of the testing datasets was lower than the chance probability, which is obviously not satisfactory performance. In order to diagnose why the single models fail, we conducted a 10-fold cross validation with the training sets of each laboratory for each single model. The training and the validation loss are plotted in Figure 4. It can be observed that although the training loss of the single models is continuously decreasing across the training process, the validation loss starts increasing shortly after around the first 10 or 20 epochs, which means the models start to overfit since then.

It can be seen that most single models performed the best on the testing dataset of lab 9, except for the three models that trained on the least size of laboratory samples, i.e., lab 1 (12), lab 3 (15), and lab 6 (20). These laboratories with small number of training samples did not perform well on their own testing sets nor did they generalize well to any other testing sets from other laboratories. The best average model performances were shown on lab 2 and lab 7, which have the largest sizes of training samples, 129 and 122, respectively. These results indicate that more numerous and diverse data are needed for a laboratory site in order to train reliable and generalizable models, at least in the contest of training RNN models.

The accuracies of single models based on test data collected from the same lab as that which provided the training data are commonly lower than the accuracies calculated using test data from different laboratories (e.g. the model developed using Lab 2 data demonstrates better test performance for the test data associated with Lab 9 than that for the test data associated with Lab 2—Figure 4). This arises through chance. It may be surprising because we might assume participants from the same lab might demonstrate similar behavior given that they were under similar experiment settings and thus the single models should perform better on their own datasets. However, the confounding factors associated with experimental biases should not be a significant factor and does not appear to be. It is worth considering that the testing sets (no matter where they are sourced) represent sets of distinct, experimental subjects whose data has never been used in training models. In other words, we expect all test subjects to be independent performers of the task and model prediction on their data can contribute to an understanding of the generalization error. Test sets from different labs contribute to our estimate of the generalization error creating a distribution. Indeed, if we observed that the test performance was highest for the lab in which the data was collected, this might suggest that local idiosyncrasies of experimental procedures were being modeled *via* the data collected. This would be an undesirable phenomenon.

### 3.3. The benefits of training collaborative models

**Figure 5:**
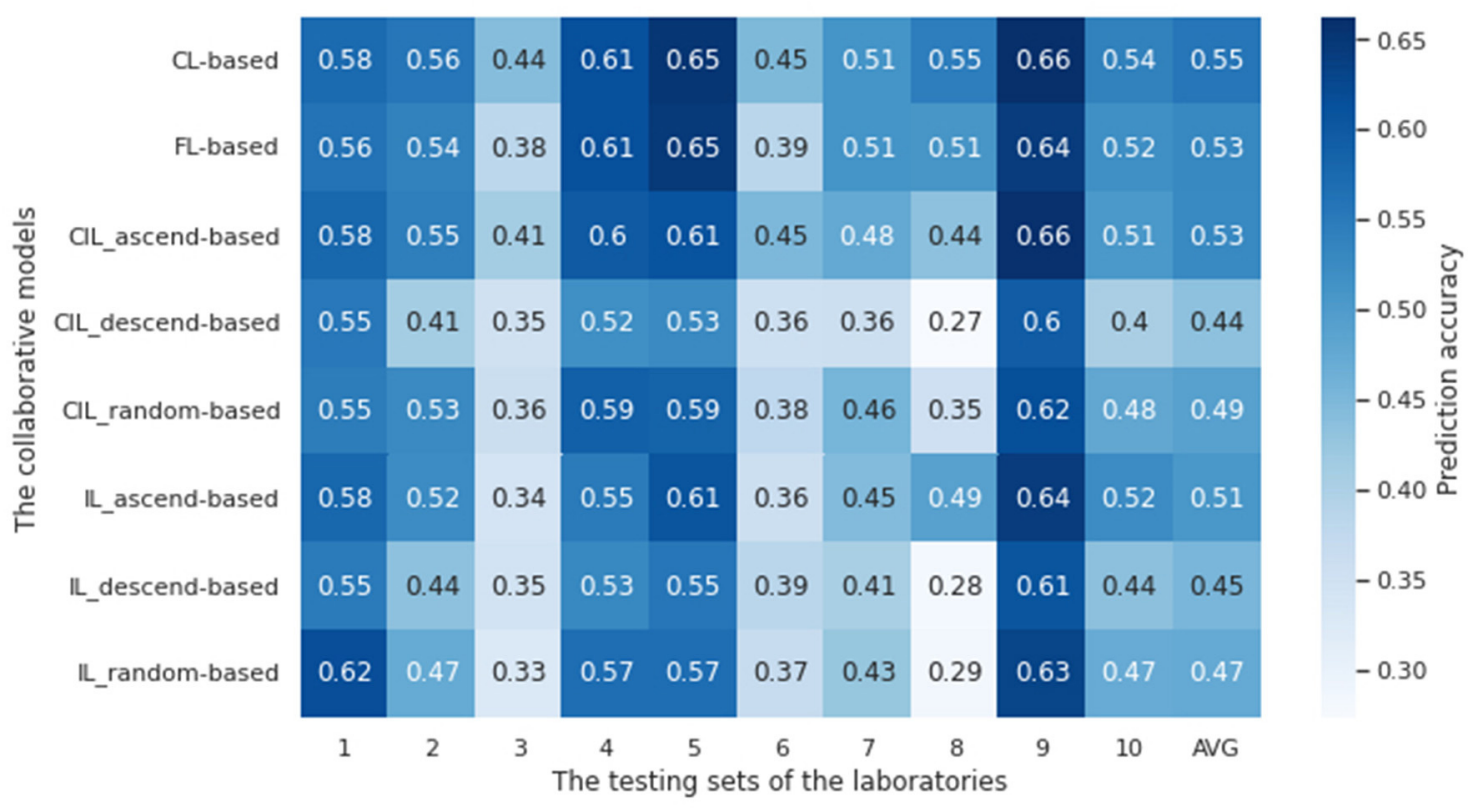
Accuracy of four collaborative models tested against testing datasets from each of the laboratories. The vertical axis represents the collaborative models trained on the training datasets from all laboratories and the horizontal axis represents the testing dataset of each independent laboratory. AVG is the average accuracy of each collaborative model performance over all laboratories. Apart from the random and descending-ordered IL and CL model, the prediction accuracy of the collaborative models is significantly improved compared to that of the single models.

In this section, we aim to demonstrate (1) the benefits of laboratory collaboration and (2) the feasibility of conducting distributed learning when data sharing barriers are present. We compared the prediction accuracy of the eight collaborative models with the single models for the first purpose. The results are illustrated in Figure 5. As a result, apart from random and descending-ordered IL and CL models, the prediction accuracy of the collaborative models was significantly improved when testing on each laboratory's data. Notably, the average performance over all laboratories of the CL-based model were improved to 55%, 6% points higher than the highest accuracy achieved by the single model of lab 2, and the corresponding increase for FL-based, CIL_ascend-based and IL_ascend-based model was 4, 4, and 3% points, respectively. Since random and descending ordered IL and CIL models did not outperform the single models, only the ascending ordered models were selected to conduct further evaluation. We will refer to these as the IL-based and the CIL-based model instead of the CIL_ascend-based and the IL_ascend-based in the following analysis for simplicity of reference. The ascending ordered IL-based and CIL-based models and the other two collaborative models were evaluated from multiple aspects for the second purpose.

### 3.4. On-policy simulation

After training the four collaborative models, we fixed the weight parameters of the models and simulated them on-policy in the task. We fed the model with the same payoff schemes as for the subjects and the models selected the decks autonomously based on what they had learned from the human behavior data. Since we trained on 491 experimental subjects from across all the studies, we simulated 491 fake agents for each collaborative model as well.

#### 3.4.1. The average probability of selecting each deck over subjects

**Figure 6:**
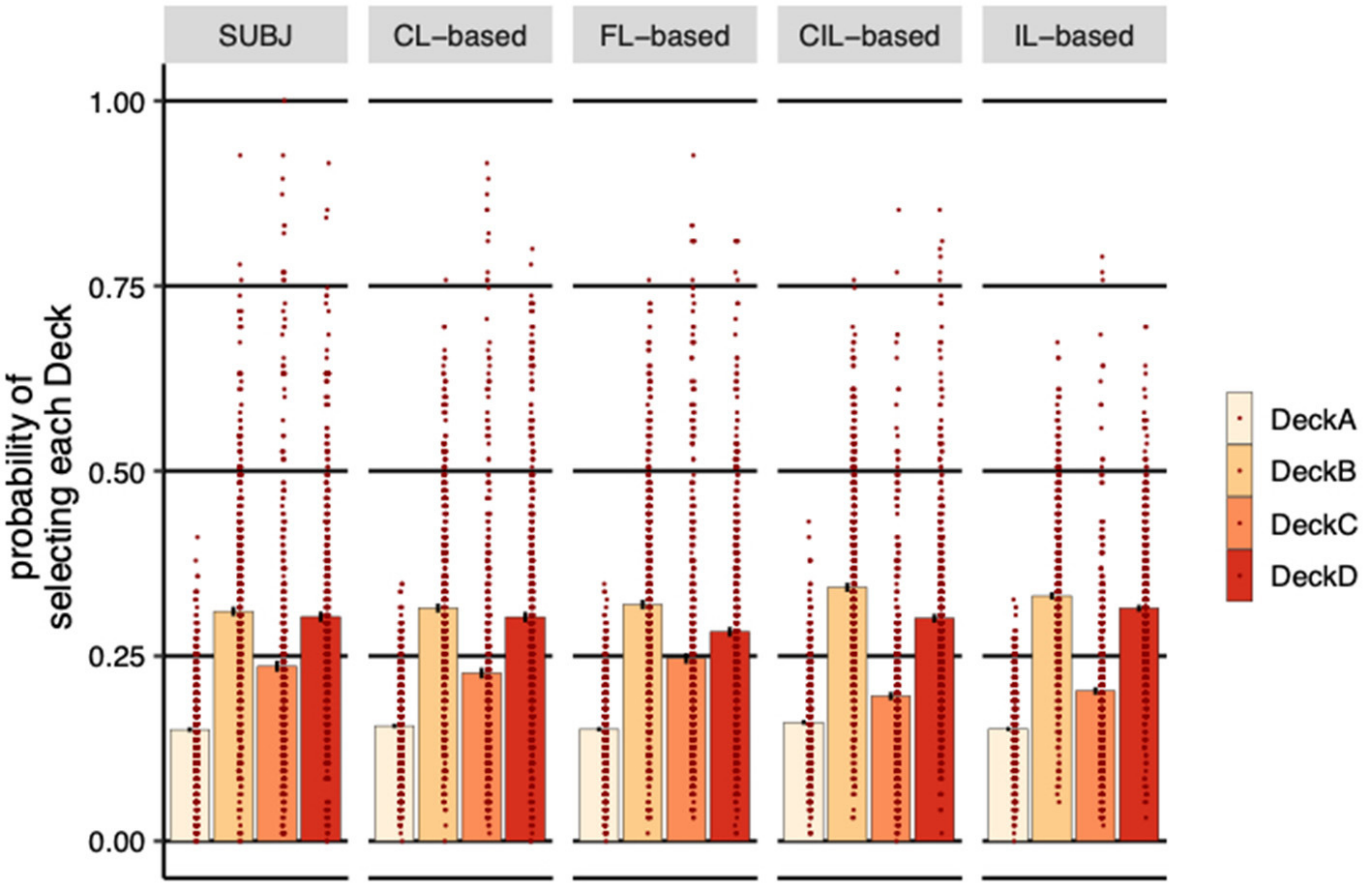
Probability of selecting each deck (averaged over subjects). Each dot represents a subject or a simulated agent and error-bars represent 1 standard error of the mean. SUBJ refers to the data of the experimental subjects; CL-based, FL-based, CIL-based, and IL-based are the on-policy simulations of the four collaborative models trained on the IGT with the same payoff scheme and the same number of trials the subjects completed, respectively. CL-based, FL-based and IL-based agents behaved more similarly to human subjects, selecting more good decks than bad decks.

The SUBJ column in Figure 6 shows the average probability of choosing each deck over all experimental subjects. All subjects selected DeckA the least compared to other Decks, which is not surprising because DeckA is the bad deck that yields negative long-term payoff and with more frequent losses. Consistent with human's choices, the probability of choosing DeckA for both centralized and distributed agents was the lowest. Neither CL-based agents (η = 0.005, *SE* = 0.004, *p* = 0.26) nor the FL-based and the IL-based agents (**FL-based:** η = 0.001, *SE* = 0.004, *p* = 0.91, **IL-based:** η = 0.001, *SE* = 0.004, *p* = 0.83) are significantly different for subjects in terms of the probability of selecting DeckA, although the CIL-based selected slightly significant more DeckA than subjects (η = 0.01, *SE* = 0.005, *p* = 0.03). FL-based agents had significant aversion to DeckD (η = −0.02, *SE* = 0.010, *p* = 0.03) and CIL-based and IL-based agents had significant preference to DeckB (**CIL-based:** η = 0.03, *SE* = 0.009, *p* < 0.001, **IL-based:** η = 0.02, *SE* = 0.008, *p* = 0.010) and significant aversion to DeckC (**CIL-based:** η = −0.04, *SE* = 0.009, *p* < 0.001, **IL-based:** η = −0.03, *SE* = 0.009, *p* < 0.001) compared to the subjects, while no significant difference was observed between CL-based agents and subjects on all of these decks. This can also be seen from the plot of the overall probabilities of selecting DeckB and DeckC where IL-based and CL-based agents are relatively higher and lower than that of the subjects, respectively, and the probabilities of selecting DeckD of FL-based agents are relatively lower.

It is worth noting that subjects prefer decks with infrequent losses, DeckB and DeckD, compared to decks with frequent losses, DeckA and DeckC, although they ended up selecting significantly more good decks (DeckC and DeckD) than bad decks (DeckA and DeckB) (η = 0.078, *SE* = 0.010, *p* < 0.001). CL-based, FL-based and IL-based agents managed to learn this strategy from the subjects' actions, selecting more good decks than bad decks (**CL-based:** η = 0.059, *SE* = 0.010, *p* < 0.001, **FL-based:** η = 0.059, *SE* = 0.011, *p* < 0.001, **IL-based:** η = 0.035, *SE* = 0.008, *p* < 0.001), whereas CIL-based agents did not learn this characteristic of behavioral pattern, the probability of selecting good decks was not significantly higher than bad decks (η = −0.006, *SE* = 0.010, *p* = 0.51).

#### 3.4.2. The fluctuation of probability of selecting each deck over trials

The analysis in the previous section suggested that CL-based, FL-based, and IL-based agents selected more good decks than bad decks, which is more consistent with the human behavioral data compared to CIL-based agents. There are multiple possible strategies that the models could follow to obtain the final proportions of each deck. Here, we aim to examine whether the models were using similar strategies to that used by subjects. We examined the fluctuation of deck preferences of the subjects and model agents over trials. We divided the 95 trials into 10 blocks of 10 trials for the first 9 blocks and 5 trials for the last block. The proportion of each deck selection in each block and the learning scores (i.e., the difference between the number of good deck selections and the number of bad deck selections) for each subject was calculated.

**Figure 7:**
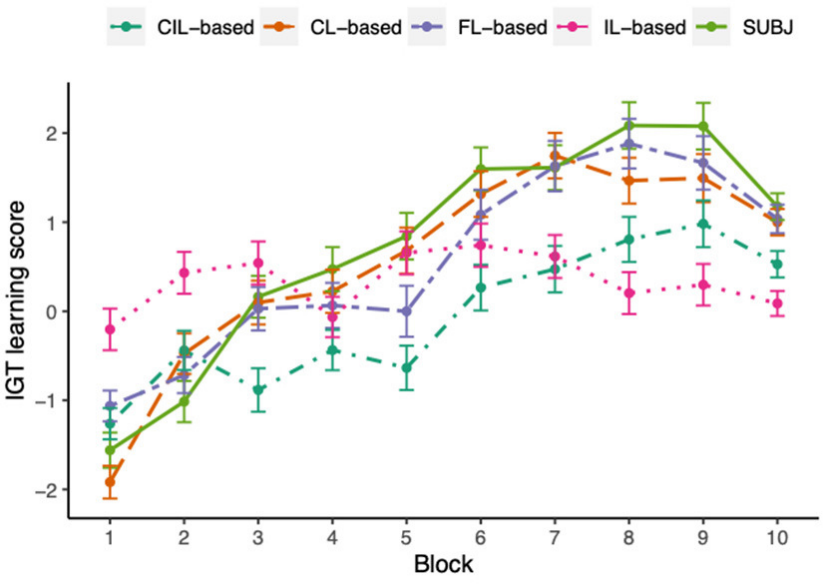
The IGT learning scores averaged over all experimental subjects or simulated model agents across 10 different blocks. Each dot represents the mean score at each block and error-bars represent 1 standard error of the mean. The learning process of human subjects, CL-based and FL-based agents is very similar to each other, overall the learning score progressively increasing over blocks. The IL-based agents are least similar to the human subjects, not presenting an obvious learning trend in the process. Although CIL-based agents demonstrated similar trends to human subjects, the magnitudes of their learning scores are always lower than that of the subjects.

Figure 7 shows the learning scores across ten blocks of the IGT. A learning process was apparent both for experimental subjects and CL-based and FL-based agents, in which the learning score progressively improved over blocks, although there was a clear dip in block 10 for subjects and FL-based agents and block 8 for CL-based agents. To quantify the differences between the subjects and the two kinds of model agents over blocks on learning scores, the repeated measures ANOVA tests were conducted in the form of 10 (blocks) × 2 (groups). The ANOVA test conducted between human and CL-based agents and human and FL-based agents revealed no significant interaction between the two factors [**CL-based:** *F*_(7.44, 7290)_ = 1.39, *p* = 0.20, **FL-based:** *F*_(7.1, 6954)_ = 1.82, *p* = 0.08], however, there was a significant main effect of blocks [**CL-based:** *F*_(7.44, 7290)_ = 67.63, *p* < 0.001, **FL-based:** *F*_(7.1, 6954)_ = 60.64, *p* < 0.001], with the interpretation that the learning scores of both subjects and CL-based and FL-based model agents varied across blocks and the CL-based and FL-based model did not behave significantly different across subjects in the learning process. The ANOVA test performed between human and the other two collaborative agents showed that the effects of blocks and group were both significant on the learning score (**CIL-based:** *F*_(7.85, 7693)_ = 47.11, *p* = 0.03, *F*_(1, 980)_ = 17.61, *p* < 0.001; **IL-based:** *F*_(7.71, 7558)_ = 22.66, *p* = 0.02, *F*_(1, 980)_ = 5.69, *p* = 0.02] and there was a significant interaction between groups and blocks on the learning score [**CIL-based:** *F*_(7.85, 7693)_ = 5.98, *p* < 0.001, **IL-based:** *F*_(7.71, 7558)_ = 15.76, *p* < 0.001]. This result suggested that the learning process of CIL-based and IL-based agents were significantly different from experimental subjects, which can also be seen from the figure, in which the learning scores of CIL-based were lower than the subjects almost over all blocks and IL-based agents did not present apparent learning trend in the process.

#### 3.4.3. The switch probability after receiving losses

**Figure 8:**
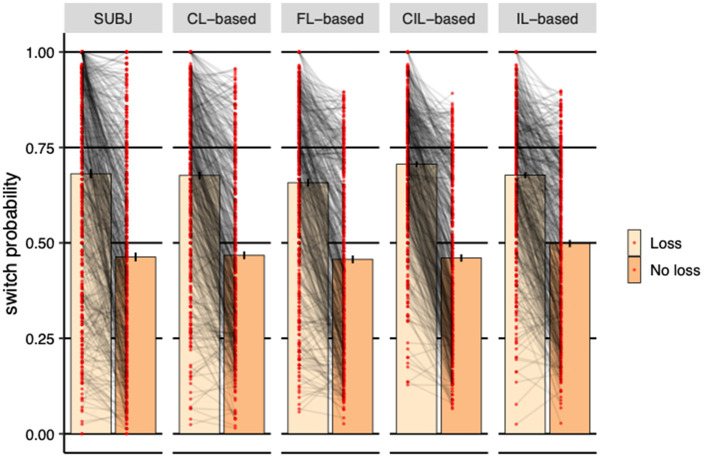
Probability of switching to a different deck based on whether received losses or not in the previous trial averaged over subjects. Each dot represents a subject or a simulated agent and error-bars represent 1 standard error of the mean. The CL-based agents demonstrated almost the same switching strategy as human subjects. FL-based agents rank second. The switching probability of IL-based agents is obviously higher than the subjects when there is no loss.

We finally investigated the immediate effect of loss on choice. The reason why reward effect is not considered here is because subjects can always get rewards no matter which deck they choose, 100 for DeckA and DeckB and 50 for DeckC and DeckD, according to the payoff scheme. Figure 8 shows the effect of receiving a loss in the previous trial on the probability of switching to the other deck in the next trial. For the experimental subjects, receiving a loss significantly increased the probability of switching to other decks (η = 0.218, *SE* = 0.010, *p* < 0.001). As the figure shows, same pattern was established by all collaborative agents (**CL-based:** η = 0.209, *SE* = 0.007, *p* < 0.001; **FL-based:** η = 0.201, *SE* = 0.007, *p* < 0.001; **CIL-based:** η = 0.246, *SE* = 0.007, *p* < 0.001; **IL-based:** η = 0.179, *SE* = 0.007, *p* < 0.001), suggesting that the strategy of avoidance to losses used by the four models was similar to that seen in the subjects' behavior. However, the switch probabilities of CL-based agents are more closer to that of the experimental subjects visually and FL-based agents rank second. The switch probability of IL-based agents was obviously higher than the subjects when there was no loss.

### 3.5. Off-policy simulation

Off-policy refers to a model which uses previous actions and payoffs to make predictions about the next action. However, the next actions actually used to simulate the models are not derived from these predictions, instead they are derived from the choices made by the human subjects. With such approach we can control the inputs the model receives and examine how they affect the predictions.

**Figure 9:**
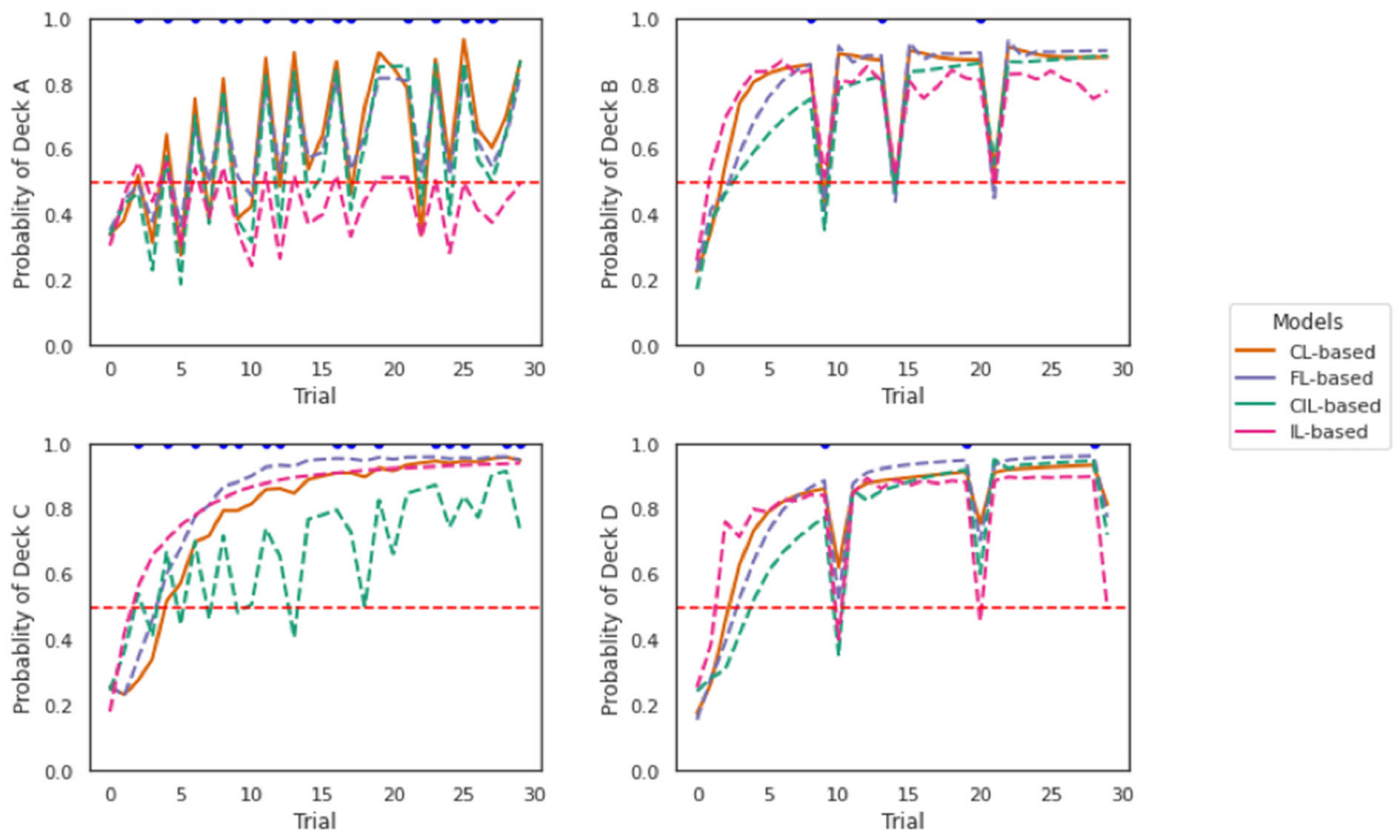
Off-policy simulations of the two collaborative models. Each panel shows a simulation of 30 trials. The four collaborative models behaved quite similarly in responding to the losses, especially the FL-based and CL-based model. CIL-based model is more sensitive to the losses given by DeckC as deeper dips are caused compared to other models. IL-based model presents least perseverance with Deck A compared with other models.

Simulations of the models are shown in Figure 9. Each panel shows a separate simulation across 30 trials (horizontal axis). For the total of 30 trials, the action that was fed into the model was DeckA (top left), DeckB (top right), DeckC (bottom left), or DeckD (bottom right) for each simulation, respectively. The reward and losses associated with these trials were the same as specified in the payoff scheme of IGT. As mentioned earlier, based on the IGT payoff scheme, the player would always receive rewards no matter which deck they choose and only the rewards magnitude varies depending on which deck they choose, while they only receive losses occasionally. We only marked trials where there were losses with blue dots in the graphs.

The four collaborative models behaved quite similarly in responding to the losses according to the plots, especially the FL-based model and the CL-based model. Receiving a loss from all decks caused a dip in the probability of staying with that deck, which showed a tendency to switch to another deck. This is consistent with the observations we obtained in Figure 8 that the probability of switching is higher when receiving a loss compared to no loss. It seems the CIL-based model was more sensitive to the losses given by DeckC as deeper dips were caused compared to other models. The dips caused by DeckB and DeckD were the deepest, which is not surprising, because DeckB and DeckD are decks with less frequent but larger losses. It is reasonable that the switch probability should be higher if you received a significant loss from that deck choice. The probability of choosing DeckA, DeckB, DeckC, and DeckD was higher than that of other decks, respectively, in each simulation. This implies sticking with the previously taken deck was an element of an deck selection strategy of the four models. We can also see that the perseverance with DeckA is not as strong as to the other three decks as the probability of selecting DeckA predicted by the two models is almost never more than 80% and this is even more obvious for IL-based model. This result is consistent with the result reported in Figure 7 where the proportion of selecting DeckA was the lowest both across subjects and trials. Interestingly, the perseverance effect to DeckC was smaller and the sensitivity to the losses of DeckC was higher for CIL-based model and relative to other models.

## 4. Discussion

The results demonstrate, as expected, that models trained on a single laboratory's dataset only, perform poorly both on their own testing sets and testing sets from other laboratories. The average prediction accuracy of the model of lab 1, which had the smallest number of subjects among studies, on ten testing datasets was 31%, only 6% higher than the chance correct probability. This result highlights the need for larger and more representative datasets and therefore emphasizes the relevance of training using collaborative models involving subjects from other studies.

Given the difficulties of sharing data across studies,institutions and jurisdictions, it is attractive to use distributed learning paradigms to replace traditional centralized data sharing to make laboratory collaboration easier. However, the decision to adopt such as an approach is only justifiable so long as the resultant model yields comparable performance with the data pooling approach. While the work presented here cannot be fully comprehensive, it presents for the first time a representative sampling of eight collaborative models, one of which based on centralized data sharing while the other seven were based on distributed learning including one FL-based, three CIL-based and three IL-based, were trained on the training datasets from all laboratories for comparison. It was found that the order of the laboratories was an important factor that influenced the performance of CIL-based and IL-based models. An ascending order based on the laboratory data-set sizes was the best strategy for both of these two paradigms and the corresponding models were selected, along with the FL-based model, to be further evaluated with the centralized model from multiple aspects. When examining their prediction capability on the unseen testing datasets the prediction accuracy was improved by 24, 24, 24, and 23% points compared to the worst single model on the same testing data-sets for CL-based, FL-based model, CIL-based and IL-based model, respectively. This highlights the value of involving larger numbers of data samples for training a deep learning model. It is also clear that FL-based and CIL-based approaches can both achieve competitive performance with CL-based model while retaining the benefit of enhanced data privacy. Another interesting finding is that all the collaborative models performed clearly better on the testing sets of lab 9, lab 5, and lab 4 and the CIL-based and IL-based models did not obviously suffer from the “catastrophic forgetting” problem as reported in the literature (Chang et al., 2018; Sheller et al., 2018, 2020). This is apparent because they did not present performance biased to the data they had mostly recently seen (i.e., lab 2 for the ascending order case, lab 1 in the descending order case, and lab 10 for the random order case).

We then evaluated the capability of the distributed model in capturing characteristics of human decision-making strategies used on the IGT in comparison with CL-based model through on-policy and off-policy simulations. In the on-policy simulation, 491 fake agents, which is the same as size as the training data-set for training the collaborative models, were generated for the four models. The proportions for choosing each deck averaged over subjects was examined first. The results revealed that CL-based agents behaved more similarly to human subjects exhibiting similar choice proportions on all decks, while the agents generated by the three distributed models had significantly different deck preferences and aversions on one or two particular decks compared to the subjects choices. This characteristic was also reflected in the fluctuation of the probability of selecting each deck over trials except that FL-based agents performed as well as CL-based agents in this comparison. The probability of choosing DeckB was almost always the highest for CIL-based and IL-based agents, whereas the probability of choosing DeckD surpassed DeckB after four blocks for experimental subjects and CL-based agents and five blocks for FL-based agents. The probability of choosing DeckC for CIL-based and IL-based agents never increased as high as that of the subjects and the other two distributed agents by the end of the game. The comparison analysis of the IGT learning scores across trials between the experimental subjects and model agents also suggested CIL-based and IL-based model were weaker in recognizing the good decks as the learning scores of these two agents did not change for IL-based agents or were always lower than the other groups throughout the task, although the overall trend of the learning score was increasing for CIL-based agents. In terms of the strategy of dealing with losses, all model agents adopted the same strategy as human subjects, i.e., switching more often when they received a loss compared to no loss. It is worth noting here that although no statistically significant weakness was identified for CIL-based and IL-based agents, the average switching probability of receiving losses was relatively higher than that of the human subjects visually, while the CL-based and FL-based agents were almost the same as the subjects on this figure. The characteristic of switching more after losses was validated again in the following off-policy simulation, where the actions fed to the model were specified by us and the models were responsible for predicting the next action to be taken for the next trial. According to the model's prediction, the probability of sticking with a deck was decreased whenever there a loss incurred. CL-based and FL-based models reacted almost the same way again in this simulation, but IL-based model demonstrated less perseverance to DeckA and the CIL-based model was more sensitive to losses compared to the other agents.

In summary, consistent with the results where FL demonstrated comparative performance on different benchmark datasets, (Dankar et al., 2019; Lee and Shin, 2020; Li et al., 2020b; Liu et al., 2021), the FL-based model in this paper achieved the best prediction accuracy and capability among the distributed learning paradigms in learning and mimicking the decision-making strategies used by the experimental subjects. Although CIL-based and IL-based models also achieved considerable prediction accuracy when compared to the CL-based model, they failed in capturing some features such as subtle deck preferences between DeckB and DeckC and learning curve shapes for the subjects. These findings can help inform researchers when developing multi-laboratory collaborations in the field of cognitive and behavioral science. It's worth speculating what deeper insights an interpretation of the parameters might reveal in terms of human choices under uncertainty. If important information describing underlying human computational elements is encoded through subtle variation in the parameter values, then the noise or uncertainty introduced through distributed learning needs to be carefully scrutinized. It suggests simulated datasets may be a useful means for determining to what extent distributed learning compromises the quality of the models developed. Importantly, the comparisons of methods introduced in this paper is informative regarding an approach to select the best technique. Additionally, emerging improvements to FL (Ozdayi et al., 2020; Wang et al., 2020) hold further promise for the narrowing of the gap in performance between centralized and federated learning.

One of the limitations of this study is that the gradient sharing scheme adopted in our distributed learning strategies is not fully protective of the privacy of the training data. Local gradients are potentially exploitable to reveal to contributory sources, an issue which is called Deep Leakage Gradients (Zhang et al., 2017; Zhu et al., 2019). However, our implementation is sufficient to demonstrate the compromises that may be necessary when adopting a distributed training approach. Future work should consider applying possible strategies to the DL methods, such as gradient pruning, to prevent deep leakage. Experiments should be conducted to make sure DL performance will not be impaired because of the application of these strategies. The other limitation of this study is that we only tested the most basic RNN model. More complex DL models, such as BiLSTM might present more competitive performance in terms of predicting human decision-making strategies.

## 5. Conclusion

Deep learning models, especially RNN models, have emerged as useful methods suitable for characterizing and even predicting human behavior in the context of computational modeling tasks. A successfully trained model generalizes well with respect to data it has not been seen before. Successful training in turn requires a large representative dataset. However, typical cognitive and behavior studies are often relatively small in terms of the numbers of subjects recruited, which limits the application of deep learning models to human behavior prediction. A natural way of obtaining bigger and more diverse data is sharing data between laboratories. Nevertheless, sharing data is never easy for the purpose of protecting personal privacy, especially when involving data that could potentially identify individuals and institutions that are internationally distributed. Distributed learning is a promising method for addressing this issue through sharing the model parameters instead of data themselves. In this study, the feasibility and reliability of applying several distributed learning paradigms to the field of cognitive studies aiming to characterize human decision-making was examined. We demonstrated that federated learning outperformed the other two distributed learning paradigms in learning the learning features of human subjects in this context. The decline of Fl-based model in terms of the capabilities of predicting and learning human behavior was not negligible and its impact depends on the subsequent use of the model. It should be highlighted while such approaches allow an ease of laboratory collaboration it does come with a model skill cost. The use of federated learning over centralized learning has the immediate advantage of keeping data confidential. We hope that the development of this techniques and emerging derivations of the approach will allow for data-private collaborative training over various subjects with different ages, education backgrounds, social status collected by different laboratories. In terms of the contribution of this work it is important to note that while distributed learning paradigms have been compared in different applications to the best of the authors' knowledge, they have never been applied to trial-by-trial human decision-making choice data. This is an important application area given the potential of such approaches for the development of digital biomarkers in the context of computational psychiatry. In that context, in which AI is potentially being used to derive a measure which could in principle directly affect a patient's treatment, the requirements for robust, reliable and accurate measurement are paramount. This requirement is increasingly enshrined in policy, regulation and law for example in the EU. Consequently an understanding of the trade-offs inherence in data privacy vs. accuracy of measurement is important and this paper demonstrates an approach for assessing this in the context of a concrete, real-world multi-center data collection effort.

## Data availability statement

The codes and data presented in the study are publicly available. This data can be found here: https://osf.io/9ekr5/.

## Ethics statement

Ethical review and approval was not required for the study on human participants in accordance with the local legislation and institutional requirements. The patients/participants provided their written informed consent to participate in this study.

## Author contributions

LZ, HV, and TW contributed to the conception and design of the study. LZ, HV, AT, and NT contributed to the model building and data analysis. LZ wrote the first draft of the manuscript. All authors contributed to manuscript revision, read, and approved the submitted version.

## Funding

This work was supported by Allied Irish Banks and Science Foundation Ireland under Grant Number SFI/12/RC/2289_P2.

## Conflict of interest

Author HV was employed by In the Wild Research Limited. The remaining authors declare that the research was conducted in the absence of any commercial or financial relationships that could be construed as a potential conflict of interest.

## Publisher's note

All claims expressed in this article are solely those of the authors and do not necessarily represent those of their affiliated organizations, or those of the publisher, the editors and the reviewers. Any product that may be evaluated in this article, or claim that may be made by its manufacturer, is not guaranteed or endorsed by the publisher.
