# Supplementary material for: A comparison of distributed machine learning methods for the support of “many labs” collaborations in computational modeling of decision making
Source: Front Psychol. 2022 Aug 25;13:943198. doi: 10.3389/fpsyg.2022.943198 (PMC9453750; doi:10.3389/fpsyg.2022.943198)
Supplement: Supplementary file 1 [file Data_Sheet_1.PDF]

Supplementary Material

1 HYPER-PARAMETER TUNING

The learning rate is set as 0.02 for all models examined in this paper. The batch size parameter for training the single models and the local models in all three distributed learning paradigms is set as the size of the training set of the training laboratory given the fact that the sizes of the training sets are not that big, not more than 100 subjects for most of the laboratories. For centralized learning where all individual training sets are pooled together, resulting a relative larger global training set that contains around 490 data points, we set the batch size as 32.

The number of epochs and the number of hidden cells (5, 10, and 20 are considered) of the RNN model for the centralized model are determined based on results of the 10-fold cross validation performed on the global training set (see figure S1). It can be seen that the validation loss is not significantly decreasing after 230 or 240 epochs and the lowest validation loss is obtained with 10 hidden cells. Thus, the number of epochs is set as 250 and the number of hidden cells is set as 10 for the centralized model.

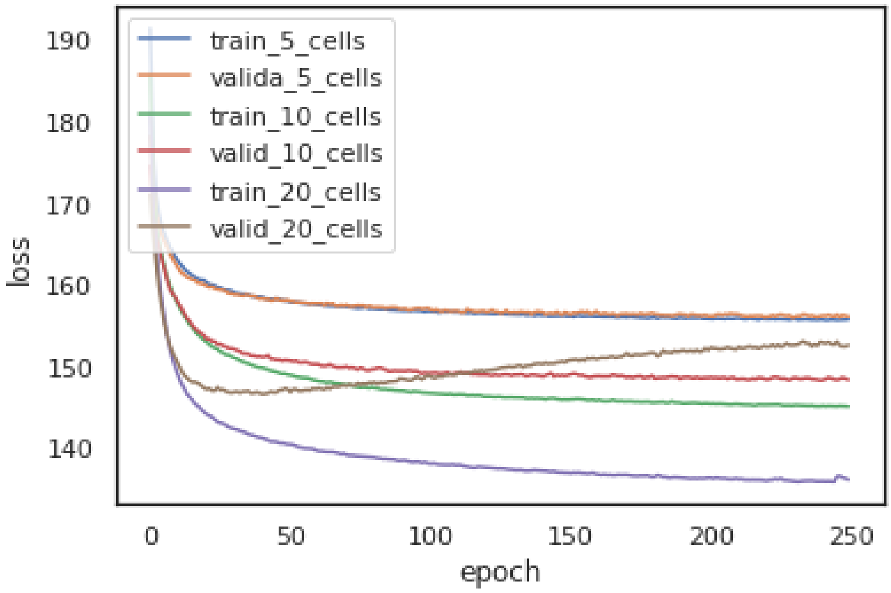

Figure S1. The average training and validation loss across 10 folds with different number of hidden cells for the centralized model. The validation loss of single models with 10 or 20 cells is even increasing as the the training progressing.

For the single laboratory models, another two most important hyper-parameter (except for the batch size and the learning rate) are the number of epochs and the number of hidden cells of the RNN model. 10-fold cross validation is performed for all single models on the training sets of each laboratory to tune these two parameter (see figure S2). The number of cells controls the level of divergence in the later stage of training. The larger the number of cells, the higher the divergence is. We chose the the number of cells to be 5. The number of epochs for all single models is set as 30 because the validation loss starts increasing since that point.

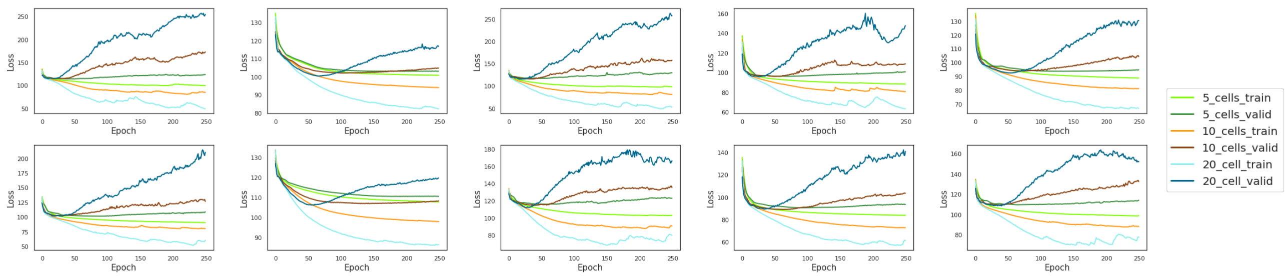

Figure S2. The average validation loss across 10 folds with different number of hidden cells for each single lab model. The turning point is approximately at 30 epochs. The larger the number of cells, the higher the divergence level is after the turning point.

For FL training, we need to determine the number of communication rounds and how many epochs of training to apply at each laboratory per round (epochs per round), which here we only consider as the same number for all laboratories and rounds. In order to create an equal playing ground for the centralized model and the distributed learning paradigms, we need to make sure that they see exactly the same number of samples in the whole training process. As a result, the multiplication of the number of communication rounds and the epochs per round for the FL training should be equal to 250, which is the number of epochs for the centralized model. The number of communication round is chosen as 5, so the epochs per round is 50. In this way, the total exposure to the training sets from all laboratories is the same as for the centralized model. Similarly, for the IL-based model, the maximum epochs for each laboratory should be set as 250. For the CL-based model, the epochs for each laboratory is considered together with the number of cycles so that their multiplication is equal to 250. In our experiment, the frequency of weight transfer is every 50 epochs, thus the number of cycles is 5. The grid search was performed for the number of cells parameter for the three distributed models. The results show that the best choice of this parameter for all of these three distributed learning paradigms is 10 cells. Additionally, the aggregation performed in federated learning is a weighted average of laboratory updates and the weightings are calculated as the ratio of the size of each laboratory training set to the size of the aggregated training set.
